# Supplementary material for: Prevalence of Selected Single-Nucleotide Variants in Patients with Neuroendocrine Tumors—Potential Clinical Relevance
Source: J Clin Med. 2022 Sep 21;11(19):5536. doi: 10.3390/jcm11195536 (PMC9573749; doi:10.3390/jcm11195536)
Supplement: Supplementary file 1 [file jcm-11-05536-s001.zip › jcm-1888978-supplementary.pdf]

Table S1. Statistics of survival analyses in the group of NET patients.

rs8005354					
	**Mean age of patients at time of admission [years ± std.dev]**	**P value**	**Median survival time [years]**	**HR (95% CI)**	**Log-rank p value**
Without minor allele	63.7 ± 11.8	0.9926	12	1.24 (0.60–2.56)	0.5822
Minor allele carriers	63.7 ± 11.4		13		
**rs2069762**					
	Mean age of patients at time of admission [years ± std.dev]	P value	Median survival time [years]	HR (95% CI)	Log-rank p value
Without minor allele	64.9 ± 10.6	0.3329	12	0.93 (0.48–1.83)	0.8381
Minor allele carriers	62.9 ± 11.9		12		
**rs3731198**					
	Mean age of patients at time of admission [years ± std.dev]	P value	Median survival time [years]	HR (95% CI)	Log-rank p value
Without minor allele	63.6 ± 11.0	0.4330	13	0.86 (0.43–1.74)	0.6550
Minor allele carriers	65.5 ± 12.4		12		
**rs1800872**					
	Mean age of patients at time of admission [years ± std.dev]	P value	Median survival time [years]	HR (95% CI)	Log-rank p value
Without minor allele	63.0 ± 11.0	0.6052	12	1.41 (0.69–2.86)	0.3310
Minor allele carriers	64.1 ± 11.9		13		

Table S2. Comparison of the risk of locoregional or distant metastasis presence depending on the presence of the rarer allele (to confirm statistical significance expected p value after the implementation of the Bonferroni correction <0.0125).

variant	*p* value
rs8005354; T>C	p=0.80637
rs2069762; A>C	p=0.04369
rs3731198; T>C	p=0.07644
rs1800872; G>T	P=0.54011
